# Bioactive Peptide of Marine Origin for the Prevention and Treatment of Non-Communicable Diseases

**DOI:** 10.3390/md15030067

**Published:** 2017-03-09

**Authors:** Ratih Pangestuti, Se-Kwon Kim

**Affiliations:** 1 Research Center for Oceanography, Indonesian Institute of Sciences (LIPI), Jakarta 14430, Indonesia; ratih.pangestuti@lipi.go.id or pangestuti.ratih@gmail.com; 2 Department of Marine-bio Convergence Science, Pukyong National University, Busan 608-737, Korea; 3 Institute for Life Science of Seogo (ILSS), Kolmar Korea Co., Seoul 137-876, Korea

**Keywords:** bioactive peptide, marine, prevention, treatment, non-communicable diseases

## Abstract

Non-communicable diseases (NCD) are the leading cause of death and disability worldwide. The four main leading causes of NCD are cardiovascular diseases, cancers, respiratory diseases and diabetes. Recognizing the devastating impact of NCD, novel prevention and treatment strategies are extensively sought. Marine organisms are considered as an important source of bioactive peptides that can exert biological functions to prevent and treatment of NCD. Recent pharmacological investigations reported cardio protective, anticancer, antioxidative, anti-diabetic, and anti-obesity effects of marine-derived bioactive peptides. Moreover, there is available evidence supporting the utilization of marine organisms and its bioactive peptides to alleviate NCD. Marine-derived bioactive peptides are alternative sources for synthetic ingredients that can contribute to a consumer’s well-being, as a part of nutraceuticals and functional foods. This contribution focus on the bioactive peptides derived from marine organisms and elaborates its possible prevention and therapeutic roles in NCD.

## 1. Introduction

**Figure 1:**
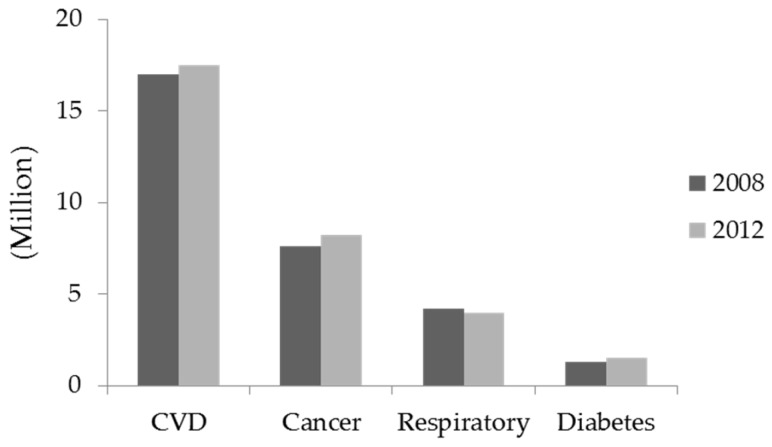
Top four cause of death attributed to non-communicable diseases in the world (References: [1,2]).

Non-communicable diseases (NCD), sometimes referred to as chronic diseases, are the leading cause of death and disability globally [1,2]. NCD are not passed from person to person, and these diseases are of long duration and slow progression. Many of the NCD are strongly associated with lifestyle-related choices (unhealthy diet, physical inactivity, and tobacco and alcohol use), and environmental and genetic factors [3]. The four main leading causes of NCD deaths are cardiovascular diseases (CVD), cancers, respiratory diseases and diabetes [4]. In 2012, CVD was responsible for around 17.5 million deaths (46.2% of NCD deaths), while cancers around 8.2 million deaths (21.7% of NCD deaths) (Figure 1).

NCD are increase rapidly poses one of the major health challenges of the 21st century. Of the 56 million global deaths in 2012, 68% or 38 million were attributed to NCD and projected to rise further worldwide. It has been predicted by the World Health Organization of the United Nations (WHO) that NCD will be responsible for a significant increase total number of deaths in the next decade. The greatest NCD increase is expected to be seen in low and middle income countries where 80% of NCD deaths occur. Notably, NCD are projected to surpass communicable, maternal, perinatal and nutritional diseases as the most common cause of death by 2030 in Africa [5]. The rapidly growing burden of NCD in low and middle income countries is not only accelerated by population aging, but also by the negative impact of globalization [2].

Recognizing the devastating impact of NCD, novel preventive and therapeutic strategies are extensively sought. Many research groups have combed both terrestrial and marine natural resources for NCD remedies [4,6,7,8]. Marine organisms are consistently exposed to biotic and abiotic pressures, which exert an influence on the organisms physiology, leading to the production of metabolites to survive and thrive [4,9]. Therefore, marine organisms are reservoirs of structurally diverse bioactive materials with numerous biological effects for human’s body. These bioactive materials include polysaccharides (agar, alginates, carrageenan, fucoidan, ulvan, laminarin, porphyran, and fulcellaran), pigments (chlorophyll, carotenoids, and phycobillins), protein and peptides, polyunsaturated fatty acids (PUFA), polyphenols, and other bioactive compounds. Among marine-derived bioactive materials, much attention has been paid to unraveling the structural and biological properties of bioactive peptides. Depending on the structural and sequence of amino acids, these peptides can exhibit diverse activities for NCD remedies, including cardio protective, antihypertensive, anticancer, anti-diabetic, and antioxidative. Not restricted to one activity, many of the bioactive peptides are multifunctional and can exert more than one of the effects mentioned. For above reasons, marine-derived bioactive peptides are considered prominent candidates for NCD prevention and treatment.

This article focuses on bioactive peptides reported from fish, mollusks, crustaceans, and seaweeds. It highlights and compiles the most relevant studies on the structural diversity of peptides found in these marine organisms and outlines their potential as candidate raw materials for the generation of bioactive peptides. Notably, their possible biological role with potential utilization as NCD prevention and remedy will be briefly discussed. Furthermore, some purification and isolation technique of marine-derived bioactive peptides will be outlined.

## 2. Marine-Derived Cardio Protective Peptides

The CVD is the leading cause of death and diseases burden in many countries [10,11]. The major independent risk factor for CVD is hypertension. In 2000, the estimated total number of adults with hypertension was nearly one billion or equal to 25% of the total adult population worldwide. The total number of adult with hypertension was predicted to increase to a total of 1.56 billion (60% of the total adult population) in 2025 [12].

**Figure 2:**
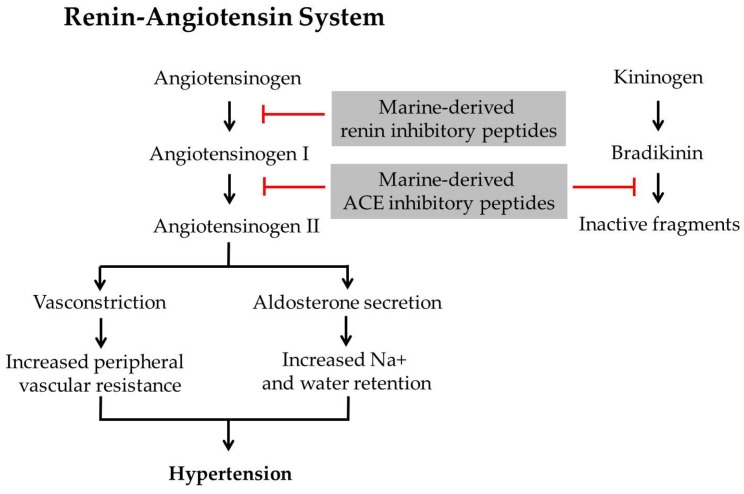
Potent renin and angiotensin-I converting enzyme inhibitory activity of marine-derived anti-hypertensive peptides.

The important regulator of blood pressure homeostasis in mammals is renin-angiotensin system (RAS). Renin (EC 3.4.23.15) converts angiotensinogen to angiotensin I, and it will be converted to biologically active angiotensin II by angiotensin-I converting enzyme (ACE, peptidyldipeptide hydrolase, EC 3.4.15.1), which ultimately leads to hypertension. In addition, ACE regulates the inactivation of bradykinin [13]. Therefore, ACE and renin inhibitor makes a positive contribution to hypertension treatment and specific inhibitors are currently used in pharmaceuticals. Synthetic hypertension drugs such as captopril, enalapril, and lisinopril are remarkably effective; however, they are known to cause adverse side effects. Hence, search for natural antihypertensive as alternative to synthetic inhibitors are of interest. Marine-derived anti-hypertensive peptides have shown potent renin and ACE inhibitory activities (Figure 2) and, therefore, potential to be used and developed as cardio protective peptides.

### 2.1. Marine-Derived Renin Inhibitory Peptides

Renin has long been recognized as the key regulator of RAS, which has an established role in controlling blood volume, arterial pressure, and cardiac and vascular function [14]. The first new class of orally active, non-peptide, low molecular weight renin inhibitors was discovered in Switzerland. The renin inhibitor was named Aliskiren (formerly CGP 60536) [15]. Afterwards, many studies have identified renin inhibitory substances derived from plant sources.

In 2012, Fitzgerald and his colleagues had successfully isolated and characterized renin inhibitory peptides derived from marine red algae *Palmaria palmata* papain hydrolysates [16]. The tridecapeptide sequence was identified as Ile-Arg-Leu-Ile-Ile-Val-Leu-Met-Pro-Ile-Leu-Met-Ala. In vivo result showed that *P. palmata* hydrolysate and tridecapeptide reduced spontaneously hypertensive rat (SHR) blood pressure when administered orally after a 24 h period. After 24 h, SHR group fed the *P. palmata* hydrolysate recorded a drop of 34 mm Hg in systolic blood pressure (SBP), while the group fed the tridecapeptide presented a drop of 33 mm Hg in blood pressure compared to the SBP recorded at time zero [17]. It was concluded that the potential active form of the peptide is dipeptides originated along the passage through gastrointestinal tract [18]. Further, *P. palmata* protein hydrolysate was formulated in wheat bread. Four percent *P. palmata* protein hydrolysate content in wheat bread did not affect the texture or sensory properties of the bread to a large degree. Interestingly, wheat bread containing the hydrolysate retained renin inhibitory bioactivity after the baking process; therefore, baked products may be one of the suitable delivery vehicles for bioactive peptides as renin inhibitor [19].

### 2.2. Marine-Derived ACE Inhibitory Peptides

It was revealed that ACE inhibitors significantly reduced the mortality of heart failure patients. Marine-derived ACE inhibitory peptides have been studied intensively and the first one was isolated from sardine by a Japanese scientist [20]. Afterwards, many other marine-derived ACE inhibitory peptides have been discovered. Up to now, more than 125 ACE-inhibitory peptides sequences have been isolated and identified from marine organisms. The potency of marine-derived ACE inhibitory peptides are normally expressed as half maximal inhibitory concentration (IC_50_) value, which is the ACE inhibitor concentration leading to 50% inhibition of ACE activity [8]. The ACE inhibition patterns of marine-derived ACE inhibitory peptides were analyzed by Lineweaver–Burk plot and the competitive inhibitions are the more frequent reported pattern compared to non-competitive inhibition [21]. Competitive inhibition means that marine-derived ACE inhibitory peptides can bind to the active site to block it or to the inhibitor binding site that is remote from the active site to alter the enzyme conformation such as that the substrate no longer binds to the active site [22].

As summarized in Table 1, peptides derived from algae, tuna, shark and salmon showed stronger ACE inhibitory activity compared to other marine organisms such as oyster, sipuncula, and jellyfish. The ACE inhibitory activity of marine-derived bioactive peptides were higher compared to ACE inhibitory peptide-derived from terrestrial food source (i.e., milk, chicken muscle and bovine) [23,24]. Marine-derived ACE inhibitory peptides are generally short chain peptides [18,25,26,27]. It was reported that amino acid residues with bulky side chain as well as hydrophobic side chains were more active for dipeptides [28]. Meanwhile, for tripeptides, the most favorable residue for the *C*-terminus was aromatic amino acids, positively charged amino acid in the middle and hydrophobic amino acid in the *N*-terminus [29]. Molecular weight is also an important factor on ACE inhibitory activity of peptides. Generally, ACE inhibitory peptides are short sequences of hydrophobic amino acids, and have low molecular weights.

Many in vivo studies in SHR and hypertensive human volunteers demonstrated that marine-derived ACE inhibitory peptides significantly reduce blood pressure. For example, bonito oligopeptide (at a dose of 3 mg/day) decreased blood pressure in human subjects with borderline or mild hypertension. More recently, the purified oligopeptide from bonito was optimized by ultrafiltration methods. The optimized bonito peptide (at a dose of 1.5 mg/day) showed anti-hypertensive effects in a double-blind, randomized, cross-over study in 61 human subjects with borderline or mild hypertension without any side-effects [47,48]. Subsequent report indicated that bonito oligopeptide played a direct action on relaxation of vascular smooth muscle in addition to the ACE-inhibitory activity [49].

Anti-hypertensive effect of peptides-derived from fish gelatin has already been reported in SHR. Peptides-derived from *O. kenojei* inhibited vasoconstriction via PPAR-c expression, activation and phosphorylation of eNOS in lungs. The peptides also involved in the expression levels of endothelin-1, RhoA, a-smooth muscle actin, cleaved caspase 3 and MAPK were decreased by SAP in lungs. SP1 (Leu-Gly-Pro-Leu-Gly-Val-Leu, molecular weight (MW): 720 Da) and SP2 (Met-Val-Gly-Ser-Ala-Pro-Gly-Val-Leu, MW: 829 Da) showed potent ACE inhibition with IC_50_ values of 4.22 and 3.09 μM, respectively [38]. Peptide from tuna and chum salmon (*O. keta*) also showed potent anti-hypertensive activity as tested in SHR [50,51]. Oral administration of tuna peptides (Gly-Asp-Leu-Gly-Lys-Thr-Thr-Thr-Val-Ser-Asn-Trp-Ser-Pro-Pro-Lys-Try-Lys-Asp-Thr-Pro, MW: 2480 Da) in SHR decreased SBP of 21 mmHg. Lee et al. (2014) demonstrated that oral administration (20 mg/kg) of chum salmon peptides showed a strong suppressive effect on SBP of SHR. They claimed that antihypertensive activity of chum salmon peptide was similar with captopril [50].

The ACE inhibitory activities of brown and red seaweed-derived bioactive peptides have been confirmed in SHR. More than one decade ago, Suetsuna et al. (2000) successfully characterized di- and tetrapeptides derived from the brown algae, *U. pinatifida* and showed that administration of those peptides in SHR significantly decreased blood pressure in SHR [25]. Marine microalgae (*C. ellipsoidea*) tetrapeptides (Val-Glu-Gly-Tyr) also showed a potent anti-hypertensive activity. Oral administration of *C. ellipsoidea* tetrapeptides at a dose of 10 mg/kg significantly decrease SBP in SHR [52].

Due to their effectiveness in regulating blood pressure, marine-derived bioactive peptides have prospective use as high quality diets for the prevention and treatment of CVD as well as other NCD. In Japan, some of the marine-derived peptides and hydrolysates have been approves as “foods for specified health uses” (FOSHU) by Japanese Ministry of Health, Labor, and Welfare. Presently, bonito oligopeptide are incorporated in blood pressure lowering capsules and sold as nutraceuticals worldwide. However, generally, marine-derived anti-hypertensive peptides are short sequences of hydrophobic amino acids, which normally give bitter taste. Therefore, to increase consumer’s acceptance, flavor manipulation needs to be used when developing marine-derived peptides as functional foods products.

## 3. Marine-Derived Anti-Cancer Peptides

Cancer is a condition of uncontrolled growth of cells which interferes with the normal functioning of the body and has undesirable systematic effects [53]. It is a dreadful NCD which increases with changing lifestyle, unhealthy diet and global warming [54]. Therefore, fruitful approaches are needed for the prevention and treatment of these diseases. Current cancer available treatments such as chemotherapy many times causing disastrous side effect; and most anticancer drugs currently used in chemotherapy are giving toxic effects to the normal cells which cause immunotoxicity and, hence, aggravate patient’s recovery [55]. In this context, a variety of ingredients of traditional medicines are being widely investigated to analyze their potential as cancer therapeutic agents. Presently, more than 60% of the used anticancer agents are derived from natural sources [56]. Although marine resources are still underrepresented in current pharmacopeia, it is anticipated that marine environment will become the invaluable source for cancer therapeutic agents in the future [57]. Many studies reported that marine-derived bioactive peptides could induce cancer cell death by different mechanisms such as apoptosis, affecting the tubulin-microtubule equilibrium, or inhibiting angiogenesis [57,58].

### 3.1. Anti-Cancer Peptides Derived from Sponges

Marine sponges (Porifera) are the oldest metazoan group, having an outstanding importance as a living fossil. There are approximately 8000 described species of sponges and perhaps twice as many un-described species. Sponges inhabit every type of marine environment, from polar seas to temperate and tropical waters and also thrive and prosper at all depths. Marine sponges have been renowned and ranked at the top with respect to the discovery of bioactive compounds with the diversity in chemical structures being related to an equally diverse pattern of activities. The chemical diversity of sponge bioactive metabolites is remarkable, including unusual nucleosides, bioactive terpenes, sterols, peptides, alkaloids, fatty acids, peroxides, and amino acid derivatives (which are frequently halogenated). In recent years, anticancer peptides have been isolated from marine sponges.

Discodermins is the first head-to-side chain novel cyclodepsipeptides isolated from marine sponge *Discodermia kiiensis.* Discodermins A–H contain 13–14 known and rare amino acids as a chain, with a macrocyclic ring constituted by lactonization of a threonine unit with the carboxy terminal. All the discodermins types are cytotoxic against murine leukemia (P388) cells, human lung (A549) cell with IC_50_ values from 0.02 to 20 μg/mL. It was demonstrated that macrolactone ring is also essential for the cytotoxic activity. Furthermore, Fusetani and co-workers (1995) reported the isolation and structure of Halicylindramides A–C, which are cyclic depsipeptides isolated from the Japanese marine sponge *Halichondria cylindrata*. Further, the structures of halicylindramide D and halicylindramide E have also been reported. Halicylindramide E is a truncated and linear version of Halicylindramide B amidated at the *C*-terminus. Compared to other type of Halicylindramide, Halicylindramide E loses cytotoxicity and shows low antifungal activity; suggesting that “head to side chain” arrangement are crucial for the bioactivity of these peptides.

Jaspamide (also known as Jasplakinolide) is a cyclic depsipeptide with 15-carbon macrocyclic ring containing three amino acid residues (l-alanine, *N*-methyl-2-bromotryptophan, and β-tyrosine). Jasplakinolide was originally isolated from the marine sponge *Jaspis johnstoni* [59]. These cyclic depsipeptides have been extensively investigated as a potential cancer therapeutic agent. Jaspamide has been demonstrated to have growth inhibitory effect on PC-3, prostate carcinoma (DU-145), and Lewis lung carcinoma (LNCaP) cells [60]. It is unique anti-cancer agents that stabilizes actin filaments in vitro, and disrupts actin filaments and induce polymerization of monomeric actin into amorphous masses in vivo. In recent years, several analogs of jaspamides have been isolated from *J. splendens* and many of them possess anticancer activity [61]. Another sponge-derived cyclic depsipeptide, Geodiomolides A, B, H and I, also showed anti-proliferative activity against breast cancer (T47D and MCF-7) cells via actin depolymerization. Geodiamolides were previously isolated and characterized from the Carribean sponge *Geodia* sp. (order Astrophorida; family Geodidae). Further experiments demonstrated that geodiamolide H induces striking phenotypic modifications in human breast cancer (Hs578T) cells [62]. Geodiamolide H decreases Hs578T cell migration and invasion which probably mediated through modifications in the actin cytoskeleton. Interestingly, Geodiamolides H was not cytotoxic for human mammary epithelial (MCF 10A) cell lines [63].

Hemiasterlins comprise a small family of naturally occurring *N*-methylated tripeptide with highly alkylated unnatural amino acids, was originally isolated from the sponge *Hemiasterella minor* (class, Demospongiae; order, Hadromedidia; family, Hemiasterllidae). Hemiasterlins act as potent tumor growth inhibitors. It was reported that Hemiasterlins exhibit antimitotic activity and thus are useful for the treatment of certain cancers. Synthetic analog of hemiasterlins, taltobulin (HTI-286) was a potent inhibitor of proliferation in 18 human tumor cell lines and had substantially less interaction with multidrug resistance protein 1 than currently used antimicrotubule agents, including vinblastine, paclitaxel, docetaxel, or vinorelbine [64]. HTI-286 and another hemiasterlin analog (E7974) are recently being evaluated in clinical trials [65].

Arenastatin A, also known as cryptophycin-24, is potent cytotoxic cyclodepsipeptide isolated from the Okinawan marine sponge *Dysidea arenaria* [66]. Arenastatin A showed extremely potent cytotoxicity against an epidermal carcinoma [67] tumor cell line. Further experiments of cryptophycin-24 showed only marginal in vivo antitumor activity, making it ineligible for further clinical trials [68]. Phakellistatins, a group of proline rich cyclopeptides, have been isolated from *Phakellia* sp. (class Demospongiae, order Axinellida). Up to now, 19 phakellistatins have been isolated [69,70,71,72]. Of all the phakellistatins, four comprise the distinctive Pro-Pro track, which represents a considerable synthetic challenge. Phakellistatin 3 represents a new type of cyclopeptide containing an amino acid unit apparently derived from a photooxidation product of tryptophan. Interestingly, all phakellistatins exhibited cancer cell growth inhibitory activities [73]. Reniochalistatins is another group of cyclopeptides rich in proline residues from an extract of a tropical marine sponge, *Reniochalina stalagmitis* Lendenfeld (class Demospongiae, order Halichondria, family Axnellidae) [74]. Recently, Zhan et al. successfully isolated reniochalistatin [75] and reported that only octapeptide (reniochalistatin) was effective inhibited growth different tumor cell lines (RPMI-8226, MGC-803, HL-60, HepG2, and HeLa). Notably, owing to conflicting reports of naturally occurring, proline-rich cyclopeptides that were initially described as having anti-proliferative activity, but subsequent synthetic samples were not active; it is premature to draw any general conclusions regarding a structure–activity relationship among the proline-rich cyclic peptides.

Mostly, anti-cancer activities of peptides-derived from sponge were investigated in vitro, therefore further detailed animal studies and clinical human trials are highly needed to evaluate the physiological anti-cancer activities of these peptides. It is important to note that sponges are susceptible to over exploitation due to their richness in bioactive compounds, hence management and conservations issue of sponge also need to be addressed. Once isolated and characterized, bioactive peptides derived from sponges can be synthesized by peptide synthesis. Synthesis of anticancer peptides derived from sponges can be used for further steps of clinical trials and may provide an alternative to the overexploitation of sponges as for medicinal purposes

### 3.2. Anti-Cancer Peptides Derived from Fish

The medicinal use of shark cartilage originated from the basic science and observational studies. Early theories regarding the use of shark cartilage for cancer stemmed from the belief that sharks are not afflicted by cancer. In 1992, William Lane published a book entitled “Sharks Don’t Get Cancer” [76]. Additionally, cartilage is often recommended by natural medicine experts for cancer, psoriasis, and inflammatory joint diseases [77]. Those traditional remedies and studies have gained attention to develop commercialized anti-cancer agents derived from shark cartilage.

Neovastat (AE-941) is a standardized liquid extract comprising the <500 kDa fraction from the cartilage of shark, *Squalus acanthias* [78]. In vitro and in vivo studies of AE-941 have demonstrated anti-tumor, anti-angiogenic and anti-inflammatory properties. AE-941 could inhibit matrix metalloproteinases (MMP)-2, MMP-9, and MMP-12, and stimulate tissue plasminogen activator enzymatic activities. AE-941 also selectively competes for the binding of vascular endothelial growth factor (VEGF) to its receptor (VEGFR), causing disruption of the signaling pathway which finally induces apoptotic activities in endothelial cells [79]. Further, AE-941 has been tested in a randomized phase III trial in patients with advanced solid tumors (prostate, lung, breast and kidney). However, the result showed that AE-941 was inactive in patients with advanced-stage cancers. AE-941 failed to meet endpoint in the phase III trial, and hence the development was stopped [80].

In 2007, Zheng et al. purified a linear polypeptide with (PG155) from the cartilage of blue shark (*Prionace glauca*). The isolated peptide could inhibit VEGF induced migration and tubulogenesis of human umbilical vein endothelial cells (HUVECs) [81]. As summarized in Table 2, anti-cancer peptides from other marine fish such as pipefish, Red Sea Moses sole, tuna, anchovy and grouper have also been isolated and purified [82,83,84,85,86]. The peptides isolated from marine fish showed anti-cancer activity in human breast cancer (MCF-7), human lung carcinoma (A549), human leukemic lymphoblasts (CCRF-CEM), hepatocellular carcinoma (HA59T/VGH), cervical cancer [87], human liver cancer (HepG2), human fibrosarcoma (HT1080), human myeloid leukemia (U937), human prostate cancer (PC-3), and oral squamous cell carcinoma (OSCC) cells. Pardaxin, a cell-penetrating peptide with cytotoxicity against cancer cells has been isolated from the marine fish Red Sea Moses sole (*Pardachirus marmoratus*) [88]. Pardaxin anti-cancer activity was mediated by apoptosis, as demonstrated by an increase in the externalization of plasma membrane phosphatidylserine and the presence of chromatin condensation. Cancer cells treated with pardaxin also showed elevation of caspase-3/7 activities, disruption of the mitochondrial membrane potential, and accumulation of reactive oxygen species (ROS) production [89]. However, compared to the snake-derived venom peptide; IC_50_ value of anti-cancer effects of marine-derived bioactive peptides is relatively higher (Table 2).

Anticancer peptide has also been isolated from half-fin anchovy (*Setipinna taty*), the peptide sequence was identified as Tyr-Ala-Leu-Pro-Ala-His. The peptide was found to be active inhibiting prostate cancer cells proliferation. Further, three modified peptide were synthesized in order to disclose the contribution of specific amino acid residue to the anti-proliferative activity. The authors concluded that hydrogen-bond formation of the guanidine moiety in arginine (R) with phosphates, sulfates, and carboxylates on cellular components was proposed to be appreciated for cell-permeation efficacy and crucial for the anti-cancer activity. However, the underlying mechanisms of anti-cancer activities are yet clarified.

The Food and Agriculture Organization of the United Nations (FAO) estimates that world global fishery capture in 2014 was 93.4 million tons, 81.5 million tons from marine waters and 11.9 million tons from inland waters [96]. These numbers are estimated to rise every year due to the increasing consumer knowledge about health benefits of fish. It was estimated that in high-risk populations, consumption of 40–60 g fish per day leads to 50% reduction in death from NCD (i.e., CVD, and cancer) [97]. Supporting those epidemiological studies, anti-cancer effects of fish-derived bioactive peptides in several cell lines also has been reported (Table 2). Unfortunately, fish consumption is very low even in some countries known for their large fish stock, such as in the north African region; hence, nutraceuticals derived from fish peptide can be develop in order to alleviate NCD. For many years, a great deal of interest has been developed by many research groups towards identification of anti-cancer peptides from fish. To develop fish-derived anti-cancer peptides as bioactive materials in food and pharmaceutical industries, large further research is needed. In addition, the potential value of fish by-product is still being ignored. It was estimated that almost half of the fish is commonly discarded to prepared seafood industrially. The amount of fisheries by-products varies depending on species, size, season, and the fishing grounds [98]. Assuming 25% of the animal weight is wasted, the total amount of waste generated from marine capture can be as high as 20.4 million tons per year. These huge amounts of fish by-product harbor useful source of anti-cancer and other bioactive peptides. Scientists should find sustainable ways to refine fish and fish by-products, and governments and industry should invest in using this marine resource in sustainable ways.

### 3.3. Anti-Cancer Peptides Derived from Urochordata

The urochordata, also known as tunicates and ascidians, have emerged as a rich source of metabolites with potent anticancer activities [99]. Chemical studies of Caribbean tunicates, *Trididemnum solidum*, led to the discovery of the didemnin depsipeptides. Of the didemnins that have been isolated, didemnin B is the most well-known member. Early studies reported that didemnin B possesses in vitro and in vivo antitumor activity against melanoma (B-16), sarcoma (M5076), prostatic, and leukemia (P388) cell lines [100,101]. Based on the significant activity and low toxicity of didemnin B in pre-clinical models, this peptide has been submitted to clinical trials, making it the first marine natural product evaluated in clinical trials [102]. Didemnin B has been tested in clinical phase I and phase II trials against several human tumors. In a clinical phase II trial, patients with non-Hodgkin’s lymphoma were given a short intravenous infusion of didemnin B every 28 days, and antitumor effects were observed [103]. Didemnin B has shown modest activity in patients with advanced pretreated non-Hodgkin’s lymphoma, and advances epithelial ovarian cancer [100,103]. Nausea, vomiting and anemia are the most frequent reported toxicities due to didemnin B. However, didemnin B clinical trials were stopped, owing to the onset of severe fatigue in patients. An analog of didemnin B that appears to be more active in preclinical models is aplidine (plitidepsin, degydrodidemnin B, DDB or aplidin). Aplidine, a cyclic depsipeptides isolated from the tunicates *Aplidium albicans*, has a pyruvyl group instead of a lactyl group in the linear peptide moiety of didemnin B [104]. Preclinical studies indicate that aplidine is active against several human tumor cell lines. Currently, aplidine has passed clinical phase I and II trials and is currently undergoing phase III trials for relapsed/refractory myeloma (NCT01102426) [105]. The exact mechanism of action of aplidine has not been fully elucidated. However, some researcher suggests that aplidine blocks the secretion of the angiogenic factor VEGF in human leukemia cells (MOLT-4) leading to the blockage of VEGF/VEGF-1 autocrine loop [106]. It has also been shown that aplidine induces a cell cycle perturbation with a block of MOLT-4 cells mainly in G1 phase of the cell cycle. Another mechanism of actions for the activity is aplidine induces cell apoptosis by inducing caspase-3 and -9 activation, cytrochrome *c* and membrane dysfunction [107]. Aplidine also induces p53-independent apoptosis in different cancer cell lines in vitro. Similar to didemnin B, aplidine also has dose-limiting toxicities, including diarrhea, dermal toxicity, asthenia, and neuromuscular.

Tamandarins A and B are two naturally occurring cytotoxic cyclic depsipeptides which are closely related to didemnin; these peptides were isolated from a Brazilian ascidian of the family Didemnidae. The structures of are similar to that of didemnin B, the molecules were found to differ only by the presence of hydroxyisovaleric acid (Hiv2), instead of the hydroxyisovalerylpropionic acid (Hip2) unit which is present in didemnins [108]. Tamandarin A showed slightly more potent cytotoxicity against pancreatic carcinoma (BX-PC3) cells, prostate carcinoma (DU145) cells, and head and neck carcinoma (UMSCC10) cells as tested in vitro. The cytotoxic effect of tamandarins has been experimentally shown, but the precise molecular mechanism of action remains uncharacterized. Another cytotoxic peptides derived from ascidian with uncharacterized molecular mechanisms is mollamide. Mollamide is a cytotoxic cyclopeptide obtained from the ascidian *Didemnum molle* and it has shown cytotoxicity against P388, A549, HT29, and monkey kidney fibroblast (CV1) cells [102]. Trunkamide A is a cyclopeptide with a tiazoline ring and structurally analogs to mollamides [109]. Trunkamide A has already undergone preclinical trials with promising antitumor effects against cell lines derived from humans, including P-388, A-549, HT-29 and human melanoma (MEL-28) cells [110].

### 3.4. Anti-Cancer Peptides Derived from Mollusks

Mollusk is one of the most diverse groups of animals on the Earth. Apart from their important ecological role and commercial value for human food, their pharmacological roles are also of notable interest. Several anti-cancer peptides have been found in mollusks. Dolastatins, a group of cytotoxic peptides, have been isolated from marine mollusks *Dolabella auricularia*, with dolastatin 10 and dolastatin 15 the most prominent [102]. Dolastatin 10 is a pentapeptide containing several unique amino acid subunits. Cytotoxic activity of dolastatin 10 against mouse lymphocytic leukemia (L1210), human promyelocytic leukemia (HL-60), human acute myelomonocytic leukemia (ML-2), human monocytic (THP-1), multiple lymphoma, small cell lung cancer (NCI-H69, -H82, -H446, and -H510) and PC-3 cells have been reported [111,112]. It has been reported that anticancer activity of dolastatin involves microtubule assembly by interacting with tubulin and blocking tubulin-dependent GTP hydrolysis [113,114]. Dolastatin 10 also affects Bcl-2 level and an increase in p53 expression [115]. However, dolastatin 10 clinical trial result was unsatisfactory; hence, dolastatin 10 was withdrawn from further trials. Another cytotoxic peptide from marine mollusk is the Keenamide A isolated from *Pleurobranchus forskalii*. These hexapeptide exhibited significant activity against the P-388, A-549, MEL-20, and HT-29 tumor cell lines [58,102,115]. Liu et al. (2012) isolated a 15 kDa linier peptides (Mere15) derived from *Meretrix meretrix* [116,117]. Mere15 inhibited the growth of leukemia (K562) cells and the cytotoxicity was related to the apoptosis induction, cell cycle arrest and microtubule disassembly [116]. Further, in vivo analysis revealed that Mere15 inhibited the growth of A549 cells xenograft in nude mice by activating intrinsic pathway [117].

Kahalalides are cyclic depsipeptides that was originally isolated from the Hawaiian marine mollusks *Elysia rufescens*. Of the seven isolated Kahalalides (A–F), Kahalalide F showed significant cytotoxic activity against cell lines and tumor specimens derived from various human solid tumors, including prostate, breast, non-small-cell lung, ovarian, and colon carcinomas [102,118]. Gonzales et al. (2003) demonstrated that cancer cells treated with Kahalalide F underwent a series of profound alterations including severe cytoplasmic swelling and vacuolization, dilation and vesiculation of the endoplasmic reticulum, mitochondrial damage, and plasma membrane rupture, suggesting that Kahalalide F induces cell death via oncosis preferentially in tumor cells. Subsequently, it was reported that ErbB3 and the downstream PI3K-Akt pathway is an important determinants of the cytotoxic activity of Kahalalide F in vitro [118]. Kahalalide F was dropped from phase II clinical trials due to a lack of efficacy despite results indicating a limited number of patients achieved a positive response. Based on the pharmacokinetic studies, it was suggested that Kahalalide F has a short half-life, which may affect its efficacy [119].

Ziconotide (formerly SNX-111, Neurex Pharmaceuticals, Menlo Park, CA, USA) is the synthetic equivalent of ω-conopeptide MVIIA, a 25-amino-acid polybasic peptide originally isolated from the venom of *Conus magus*, a marine snail [120]. Ziconotide is an analgesic agent administered intrathecally and has been for almost one decade for the treatment of chronic cancer pain [121]. However, the use of ziconotide can induce several and sometimes serious adverse events. Hence, a low initial dosage followed by slow titration is recommended to reduce serious adverse events.

### 3.5. Anti-Cancer Peptides Derived from Cyanobacteria

Cyanobacteria (blue-green algae) are a very old and diverse group of photosynthetic, prokaryotic organisms that produce a variety of secondary metabolites with various biological activities, including phenols, peptides, alkaloids or terpenoids [122]. Cyclic depsipeptides, grassypeptolides D and E, have been isolated from the marine cyanobacterium *Leptolyngbya* sp. [123]. These peptides have shown cytotoxic effect against mouse neuroblastoma (N2A) and HeLa cell line, which was confirmed by MTT cell viability assay. *Lyngbya majuscula*, a benthic filamentous marine cyanobacterium, has been extensively studied and has produced more than 250 compounds with diverse structural features. This diversity is in part attributable to the fact that a major theme in *L. majuscula* biochemistry relies on the production of metabolites via polyketide synthases and nonribosomal peptide synthetases within specialized biosynthetic pathways. Malyngamide 4, somocystinamide A, and hectochlorins are potent anti-cancer lipopeptides isolated from *L. majuscula* [124,125,126]. Hectochlorins have been reported to be strong actin-disrupting agents. Hectochlorin showed great anti-proliferative activity against colon, melanoma, ovarian, and renal cancer cells [127]. Shaala et al. (2013) demonstrate that malyngamide A inhibited proliferations of A549, HT29, and breast adenocarcinoma (MDA-MB-231) cells cultured in vitro. Another lipopetide isolated from *L. majuscula*, Somocystinamide A showed potent cytotoxicity against N2A cells. Further, Somocystinamide was found as potent apoptosis inductor in a number of tumor cell lines and angiogenic endothelial cells via intrinsic and extrinsic pathways, but the more effective mechanism is the activation of caspase 8 [126]. Apratoxin A is a cyclodepsipeptide isolated from a *L. majuscula*. This peptide showed anti-proliferative activity in KB and LoVo cancer cells. Apratoxin A mediates its anti-proliferative activity through the induction of G1 cell cycle arrest and an apoptotic cascade, which partially initiated through antagonism of FGF signaling via STAT3 [128].

The blue-green colored pigment-protein complex, c-phycocyanin, isolated from marine cyanobacteria *Agmenellum quadruplicatum*, *Mastigocladus laminosus*, *Oscillatoria tenuis* appeared to be a potent activator of pro-apoptotic gene and downregulator of anti-apoptotic gene expression [129]. Transduction of apoptosis signals resulting apoptosis of HeLa cells in vitro [130]. Further, apoptosis features such as cell shrinkage, membrane blebbing, nuclear condensation and DNA fragmentation were observed in A549 and HT29 treated with c-phycocyanin [131].

Cyanobacteria possess several advantages to be developed as nutraceuticals for the prevention and treatment of cancer and other NCD. The advantages of cyanobacteria include simple growth requirement, ease of genetic manipulation, and attractive platforms for carbon neutral production process [132]. However, it should be noted that some cyanobacteria produce cyanotoxins, therefore an appropriate regulatory framework should be developed for pharmaceutical and nutraceutical products from cyanobacteria to ensure that safety and quality standards are met.

## 4. Marine-Derived Antioxidant Peptides

In addition to the general risk factors in the development of NCD, free radicals are also known to play a significant role in NCD. Marine-derived protein, protein hydrolysates, peptides and amino acids have been shown to have significant antioxidant effects. Marine organisms are probably the most extensively studied as an important source of antioxidants. Antioxidant activity of marine organisms has been determined by various in vitro and in vivo methods, such as 2,2-diphenyl-1-picrylhydrazyl (DPPH), peroxide, hydroxyl and superoxide anion radical scavenging activities which have been detected by electronspin resonance spectroscopy method as well as intra cellular free radical scavenging assays, such as DNA oxidation, ROS scavenging, membrane protein oxidation and membrane lipid oxidation [133]. Many studies reported that proteins from marine organisms exhibit potent antioxidant activity; however, in many cases, peptide fractions or protein hydrolysates showed greater antioxidant activity. These suggest that peptides play a significant role in antioxidant actions of marine proteins. Therefore, many individual bioactive peptides responsible for antioxidant activity of marine protein or protein hydrolysates were then purified and identified. Marine-derived peptides have varied antioxidant activities depending on the structure. The peptide structure including the size and amino acid sequences were influenced by the protein sources and extraction conditions. As an example, clam peptides, isolated from body or viscera of clam (*Meretrix casta*) protein hydrolyse with three different enzymes such as trypsin, pepsin and papain resulted in different DPPH radical scavenging activities, ranging from 9.1% to 82.5% and reducing power ranging from 0.1 to 0.7, measured as the ability of the hydrolysate to reduce iron (III) [134]. Rajapakse et al. (2005) identified four different molecular weight peptides from giant squid mussel by employing ultrafiltration membrane with three different molecular weight cut off membranes (10, 5 and 3 kDa). Lower molecular weight peptide was found to possess stronger antioxidant activity compared to the higher molecular weight peptides. They assumed that lower molecular weight improves contact ability with membrane lipids and or permeability [135]. Further, it is believed that aromatic amino acid and histidine act positively as direct radical scavengers within peptide sequences. The presence of aromatic amino acids in the structure of a peptide is an advantage in this regard because they can donate protons easily to electron-deficient radicals and, at the same time, maintain their stability via resonance structures. Hence, it can be speculated that difference in scavenging activity could be due to the molecular weight or the specific arrangement of amino acid residues in the peptide sequence [13].

In addition to marine peptides, marine processing by-products have also been explored for production of proteins, peptides, and hydrolysates with antioxidant potentials [136]. Purification of antioxidant peptides derived from marine by-product using enzymatic hydrolysis has been in practice during recent years. Antioxidant peptides derived from marine processing by-product were found to possess strong antioxidant activity in linoleic acid model [137,138]. Himaya et al. (2012) demonstrated that peptide isolated from Japanese flounder skin gelatin could protect against cellular oxidative damage. Some peptides derived from marine processing by-product were found to possess strong activity to inhibit lipid peroxidation in linoleic acid models. This activity was attributed to the ability of peptide to interfere propagation cycle of lipid peroxidation and there by slowing radical mediated linoleic acid oxidation. Hydrophobic amino acids in peptide sequences may contribute to peroxidation inhibition by increasing the solubility of peptide in lipid and thereby facilitating better interaction with radical species [139]. Position of hydrophobic amino acid, Leu at the *N*-terminus of the peptide sequences has been shown to increase the interaction between peptides and fatty acids. More importantly, hydrophobic peptides can protect macromolecule oxidation by donating photons to reactive radicals [13,140]. Moreover, the activity of histidine containing peptides has also been reported to act against lipid peroxidation. In addition, Shahidi and Zhong (2008) reported that in the case of tripeptide, tripeptides containing 2 tyrosine units had higher capacity than those containing 2 histidine units in inhibiting linoleic acid oxidation. Later, it was reported that histidine-containing peptides can act as metal chelator, active oxygen quencher, and hydroxyl radical scavenger, thus contributing to the antioxidant activity of the protein hydrolysate and peptide.

Epidemiological studies show that a diet rich in antioxidants is associated with low prevalence of NCD, longevity and good health. Therefore, researchers are continually seeking for a good source of diet with potent antioxidant ability as an alternative for the dietary supplements and food. Bioactive peptides of marine origin have the potential to subside the biochemical imbalances induced by the formation of free radicals, and many of these peptides have been viewed as promising agents for the prevention and treatment of NCD. One of the commercially available products from marine organisms to reduce oxidative stress is Fortidium Liquamen, a hydolyzed skin of white fish (*Molva molva*) [141]. Based on those collective findings, it may be assumed that marine-derived bioactive peptides is a healthy choice to strengthen the body’s fight against oxidative stress and other related NCD.

## 5. Anti-Diabetic and Hypocholesterolemic Effects of Marine-Derived Bioactive Peptides

Metabolic disorders comprise a collection of health disorders that increase the risk of morbidity and loss qualify of life, these includes diabetes and obesity. Marine-derived proteins and their peptides exert anti-diabetic effects. Zhu et al. (2010) have reported that treatment with oligopeptides from marine salmon skin modulated type 2 diabetes mellitus-related hyperglycemia and β-cell apoptosis in rats induced by high fat diet and low doses of streptozotocin. The anti-diabetic effect of salmon skin-derived oligopeptides was mediated by down-regulation of type 2 diabetes mellitus-related oxidative stresses and inflammation, which then protect the pancreatic β-cells from apoptosis [142].

A marine collagen peptide (MCP) isolated from wild marine fish caught from the East China Sea has shown anti-diabetic effects in patients with or without hypertension [143]. The levels of free fatty acid, hs-CRP, resistin and prostacyclin were decreased significantly following MCP treatment, indicating that MCP could offer protection against diabetes and hypertension by affecting levels of molecules involved in diabetic and hypertensive pathogenesis. Further, it was confirmed that MCP modulates glucose and lipid metabolism in patients with type 2 diabetes mellitus [144]. It was demonstrated that MCP is a peptide mixture containing two to six amino acid residues in length with molecular weight 100–800 Da. Unfortunately, the amino acid sequence of MCP is not elucidated yet. Peptide possess anti-metabolic disorder are generally low molecular weight (500–800 Da) [145]. Peptide sequence also plays an important role in anti-diabetic and anti-obesity effects. Generally, anti-diabetic and anti-obesity peptides are hydrophobic. Such a hydrophobic peptide is envisaged to be able to cross (biological) membranes. Vernaleken et al. (2007) described that specific functional tripeptide fragments (i.e., “Gln-Cys-Val” and “Gln-Cys-Pro”) are potent inhibitors of monosaccharide-dependent exocytotic pathway of Na^+^-d-Glucose co transporter SGLT1. The specific peptide sequence may influence negatively specific nutrient transporters/receptors in vivo which further lead to posttranscriptional down regulation of nutrient transporters and reduction of body weight [146]. It was also reported that high amounts of Gly amino acids in marine-derived proteins could contribute to an increase in fecal cholesterol and/or bile acid excretion, thus contributing to improvement in plasma lipid variables [147]. In addition, low molecular weight peptides derived from Salmon rich in Gly significantly alleviated obesity-linked inflammation. Many studies have shown that pro-inflammatory mediators including tumor necrosis factor-α (TNF-α), interleukin-1β (IL-1β) and interleukin-6 (IL-6) are increased during obesity and diabetes. The suppression of these pro-inflammatory mediators may decrease the risk of developing metabolic disorders-associated inflammation and insulin [148].

Hyperlipidemia, particularly hypercholesterolemia, is an obesity related condition common in diabetic patients, and also one of the most important risk factors contributing to the development of NCD. Natural extracts with cholesterol-lowering effect have been explored for their potential in prevention and treatment of hypercholesterolemia. In vivo study showed that protein derived from microalgae (*Spirulina platensis*) c-phycocyanin, plays a crucial role in the hypocholesterolemic activities [149]. In addition, Colla et al. (2008) demonstrated that *Spirulina platensis* when added in rabbit feed for 30–60 days reduced the levels of total cholesterol, high-density lipoprotein and triacylglycerols [150].

Current food environments are unhealthy which dominated by energy-dense, nutrient-poor processed food products which are widely available and relatively inexpensive [151]. These seem to create a supply-side “push” effect on unhealthy diets which is the prevailing driver of population unhealthy weight gain and NCD. To reduce hypercholesterolemic, diabetes, and other diet-related NCD, there needs to be a central focus on creating “healthy food environments” which shift population diets, especially those of socially disadvantaged populations, towards healthy diets. Marine-derived bioactive peptides have excellent potential as functional food ingredients to reduce NCD as they possess advantageous physiological effects, with medicinal characteristics and added health benefits such as anti-diabetic and hyocholesterolemic activities.

## 6. Future Perspectives of Marine-Derived Bioactive Peptides

Successful characterization of marine-derived bioactive peptides and investigations of their cardio protective, antihypertensive, anticancer, anti-diabetic, and anti-oxidative effects suggest their promising future for NCD. However, current marine peptides are still unable to meet the design parameters for drugs for NCD due to their low metabolic stability, low membrane permeability, and their high costs of manufacture [152]. Therefore, marine-derived bioactive peptides can be administered using different delivery vehicles such as functional food and or nutraceuticals. In order to be used as ingredients in food products, different studies should be carried out to determine if bioactivity of marine peptides is maintained after manufacturing and cooking processes. For example, wheat bread containing the hydrolysate from red algae retained renin inhibitory bioactivity after the baking process [19]. Furthermore, biological effect of marine-derived peptides is strongly influenced by their bioavailability, which is predominantly determined by their susceptibility to degradation into inactive fragments by digestive enzymes peptidase and intestinal absorption. Bioavailability should be taken into account when developing food and beverages products containing marine-derived bioactive peptides for the prevention and treatment of NCD.

Bioavailability of peptides can be defined as the quantity that passes through the cell membranes in the intestine and is available for action within the cells [153]. Bioavailability of peptides are generally affected by physicochemical properties of the peptides such as molecular size, charge, sequence, and solubility; smaller peptides are transported across the enterocytes through intestinal-expressed peptide transporters, whereas oligopeptides may be absorbed by passive transport through hydrophobic regions of membrane epithelia or tight junctions [154]. Many studies demonstrated that marine-derived peptides are mostly peptides of small molecular weights, especially tripeptides from marine algae and small oligopeptides. These small molecular weight peptides are too small for the substrates of digestive proteases, and therefore they have high resistance to gastrointestinal digestion and are easily to be absorbed. In addition, small molecular weight peptides are convenient and cheaper to be synthesized through chemical method. Thus, chemical synthesis can be used to produce large quantities of marine-derived bioactive peptides to be used in functional foods and pharmaceuticals to meet the needs for NCD remedy.

Several studies have demonstrated the bioavailability of marine-derived bioactive peptides for the treatment of NCD using both animal models and human volunteers. For example, long-term oral administration of peptides derived from jellyfish reduced systolic blood pressure and diastolic blood pressure of the renovascular hypertension rats [155]. Interestingly, these bioactive peptides affected the production of Angiotensin II only in kidney but not in plasma. In addition, Lee et al. (2010) demonstrated that oral administration of peptide-derived from tuna frame significantly reduced systolic blood pressure and diastolic blood pressure in spontaneously hypertensive rats. That information provides basic information that peptide-derived from tuna frame show stability against gastrointestinal proteases and original peptide sequences that displayed anti-hypertensive activity are delivered to the cellular sites of action. These provide evidence that marine-derived bioactive peptides can be used for the preparation of oral treatment for blood pressure homeostasis which further protects cardiovascular system.

Up to now, many marine peptides are unable to meet the requirements for food (e.g., taste, bioavailability, or stability). Bitterness of some marine-derived peptides is an undesirable property, which should be reduced during food, beverages and or pharmaceuticals production. Marine-derived bioactive peptides hosting residues with hydrophobic side chains have a distinct bitter taste. Therefore, further studies on controlling these properties are needed. These can be achieved by several methods including chemical or physical modifications of the peptides (i.e., microencapsulation, and quantitating the bitter taste relationship). Microencapsulation not only increases consumer’s acceptance, but also ensures that the marine-derived peptide sequences that displayed bioactivity are conserved and delivered to the cellular sites of action in NCD. Further, microencapsulation will enhance their stability and absorption.

In order to develop food and beverages product containing marine-derived bioactive peptides, methods must be developed to enhance their availability and bioactivity. Bioactive peptides can be obtained from marine organisms by organic solvent extraction, fermentation and enzymatic hydrolysis by proteolytic enzymes. In food industries, the last methods are more preferred due to the lack of residual organic solvents or toxic chemicals in the products and or microbial residue. Notably, physico-chemical conditions of the reaction media, such as temperature and pH of the protein solution, must then be adjusted in order to optimize the activity of the enzyme used. Further, to obtain desired molecular weight and functional properties of marine-derived bioactive peptides, a suitable method is the use of an ultrafiltration membrane system. This system has the main advantage that the molecular weight distribution of the desired peptide can be controlled by adoption of an appropriate ultrafiltration membrane.

The number of marine organism’s consumption is estimated to rise each year due to the increasing consumer knowledge about their health benefit effects. Marine organisms are viewed as “natural and healthy” by consumers, and this promotes a positive response in consumers, who often regard natural entities. Therefore, marine organisms may be considered a consumer friendly source of functional foods which may use to prevent and treat NCD. Last but not least, scientists should work out sustainable ways to refine bioactive peptides derived from marine organisms, and develop food and pharmaceuticals products to alleviate NCD.

## 7. Conclusions

Many studies have shown that marine-derived bioactive peptides possess remarkable activities relevant to the prevention and treatment of NCD. The possibilities of designing new functional foods, nutraceuticals, and pharmaceuticals derived from marine bioactive peptides for the prevention and treatment of NCD are promising. While much information is available on biological activities of marine-derived bioactive peptides, future studies should be directed towards evaluation of bioavailability in human subjects as well as clinical trials. In addition, safety and quality standards of marine-derived peptides-based products should be evaluated prior to commercialization.

## Figures and Tables

**Table 1:** ACE inhibitory activity of marine-derived bioactive peptides.

Source	Extraction	Sequence	Inhibition (IC_50_)	References
Seaweed (*Undaria pinnatifida*)	Hot water extraction; Chromatography	Ile-Tyr	2.7 μM	[18]
	Enzymatic hydrolysis (Protease S); Chromatography	Ile-Trp	1.5 μM	[30]
Seaweed (*P. yezoensis*)	Chromatography	Ala-Lys-Tyr-Ser-Tyr	1.52 μM	[31]
Microalgae (*Spirulina platensis*)	Enzymatic hydrolysis (Pepsin); Chromatography	Ile-Ala-Pro-Gly	11.4 μM	[32]
Yellowfin tuna (*Neothunnus* *macropterus*)	Chromatography	Pro-Thr-His-Ile-Lys-Trp-Gly-Asp	2 μM	[33]
Skipjack tuna (*Katsuwonus pelamis*) bowels	Chromatography	Leu-Arg-Pro	1 μM	[34]
Alaska Pollack skin (*Theragra chalcogramma*)	Enzymatic hydrolysis (serial protease); Chromatography	Gly-Pro-Leu	2.6 μM	[35]
Chum salmon (*Oncorhynchus keta*) muscle	Enzymatic hydrolysis (Thermolysin); Chromatography	Val-Trp	2.5 μM	[36]
Pink salmon (*Oncorhynchus gorbuscha*)	Enzymatic hydrolysis (papain); Chromatography	Ile-Trp	1.2 μM	[37]
Skate skin (*Okamejei* *kenojei*)	Enzymatic hydrolysis (alkalase/protease); Chromatography	Met-Val-Gly-Ser-Ala-Pro-Gly-Val-Leu	3.09 μM	[38]
Small-spotted catshark (*Scyliorhinus canicula*)	Enzymatic hydrolysis (Trypsin, subtilisin); Chromatography	Val-Ala-Met-Pro-Phe	0.44 μM	[39]
Pelagic thresher (*Alopias pelagicus*) muscle	Enzymatic hydrolysis (thermolysin); Chromatography	Ile-Lys-Trp	0.54 μM	[26]
Marine shrimp (*Acetes chinensis***)**	Enzymatic hydrolysis (Protease); Chromatography	Ile-Phe-Val-Pro-Ala-Phe	3.4 μM	[40]
	Fermentation; Chromatography	Asp-Pro	2.15 μM	[41]
	Enzymatic hydrolysis (Pepsin); Chromatography	Leu-His-Pro	3.4 μM	[42]
Izumi shrimp (*Plesionika izumiae* Omori, 1971)	Enzymatic hydrolysis (Protease); Chromatography	Ser-Thr	4.03 μM	[43]
Jellyfish (*Rhopilema* *esculentum*)	Enzymatic hydrolysis (pepsin, papain); ultrafiltration; Chromatography	Gln-Pro-Gly-Pro-Thr	80.67 μM	[44]
Sipuncula (*Phascolosoma esculenta*)	Enzymatic hydrolysis (Pepsin); Chromatography	Ala-Trp-Leu-His-Pro-Gly-Ala-Pro-Lys-Val-Phe	135 M	[45]
Pearl oyster (*Pinctada fucata martensii)*	Enzymatic hydrolysis (Pepsin); Chromatography	Ala-Leu-Ala-Pro-Glu	167.5 μM	[46]

**Table 2:** Anti-cancer effects of bioactive peptides derived from marine fish and other organisms.

Name	Source	Anti-Cancer Activity	References
Neovastat (AE-941)	Spiny dogfish shark (*Squalus acanthias*)	Inhibition of metastatic activity on HUVEC, BAEC cells; inhibition of matrix metalloproteinase; Anti-angiogenic effects; Pro-apoptotic on BAEC cells	[78,90]
Pardaxin	Red Sea Moses sole (*Pardachirus marmoratus*)	Pro-apoptotic on HT1080 (IC_50_: 14.52–15.74 μg/mL), HeLa, OSCC cells	[89,91,92,93,94]
PG155	Blue shark (*Prionace glauca)*	Anti-angiogenic effects on HUVECs	[81]
Syngnathusin	Pipefish (*Syngnathus acus)*	Pro-apoptotic on A549 (IC_50_: 84.9 μg/mL), and CCRF-CEM (IC_50_: 215.3 μg/mL), cells	[86]
Epinecidin-1	Grouper (*Epinephelus coioides*)	Anti-angiogenic effects on A549, HA59T/VGH, HeLa, HepG2, and HT1080 cells Pro-apoptotic on U937 cells	[82,83]
PAB 1; PAB2	Long tail tuna (*Thunnus tonggol*)	Pro-apoptotic on MCF-7 cells (IC_50_: 8.1; 8.8 μM)	[84]
YALRAH	Half-fin anchovy (*Setipinna taty*)	Pro-apoptotic on PC-3 cells (IC_50_: 11.1 μM)	[85]
Rusvinoxidase	Venom of *Daboia russelii russelii*	Pro-apoptotic on MCF-7 cells (IC_50_: 83 nM)	[95]

## Abbreviations

The following abbreviations are used in this manuscript:

ACE	Angiotensin converting enzymes
CVD	Cardiovascular disease
DPPH	2,2-diphenyl-1-picrylhydrazyl
FAO	Food and Agriculture Organization
FOSHU	foods for specified health uses
IC50	half maximal inhibitory concentration
IL-6	interleukin-6
IL-1β	interleukin-1β
MCP	marine collagen peptide
MW	Molecular weight
NCD	Non communicable diseases
PUFA	polyunsaturated fatty acids
RAS	renin-angiotensin system
ROS	reactive oxygen species
SHR	spontaneously hypertensive rat
SBP	systolic blood pressure
TNF-α	tumor necrosis factor-α
WHO	World Health Organization
